# Total ureteral replacement with a small intestinal graft as a reconstructive stage of combined treatment for recurrent colorectal cancer: case report

**DOI:** 10.3389/fonc.2026.1767642

**Published:** 2026-06-01

**Authors:** Sergey K. Efetov, Maksim V. Volgin, Aleksandra Y. Koziy, Igor V. Fokin, Camilla R. Azilgareeva, Nina B. Paramonova, Mikhail E. Enikeev, Airazat M. Kazaryan

**Affiliations:** 1 Department of Faculty Surgery No. 2, I.M. Sechenov First Moscow State Medical University, Moscow, Russia; 2 Institute of Urology and Human Reproductive Health, I.M. Sechenov First Moscow State Medical University (Sechenov University), Moscow, Russia; 3 Institute of Clinical Morphology and Digital Pathology for Diagnostic Work, I.M. Sechenov First Moscow State Medical University (Sechenov University), Moscow, Russia; 4 Department of Gastrointestinal and Pediatric Surgery, Oslo University Hospital, Oslo, Norway; 5 Department of Surgery, Østfold Hospital Trust, Grålum, Norway; 6 Department of Abdominal Surgery, Yerevan State Medical University after M.Heratsi, Yerevan, Armenia

**Keywords:** colorectal cancer, colorectal cancer metastases, ileoureteroplasty, lymph node dissection, ureteral reconstruction

## Abstract

**Introduction:**

We present a clinical case highlighting total ureteral replacement with a small intestinal graft as a key reconstructive component in the surgical management of recurrent colorectal cancer.

**Case description:**

A 48-year-old female patient previously underwent low anterior rectal resection with D2 lymph node dissection (pT2N0M0, 2018). Four years later, she presented with clinical signs of obstruction in the middle third of the left ureter. Imaging revealed a mass involving the ureteral wall and left psoas muscle, alongside left adnexal abnormalities. Surgical treatment was staged and included en bloc resection of the recurrent mass with removal of the left ureter, adnexectomy, and segmental colectomy. In the remission phase, ureteral reconstruction was performed using a small intestinal graft, followed by transverse colon mobilization and the creation of a stapled transverse-rectal anastomosis.

**Discussion:**

This case illustrates how timely, organ-preserving reconstruction – integrated into comprehensive oncological surgery – can improve both survival and quality of life in patients with locally advanced recurrent rectal cancer.

## Introduction

Colorectal cancer (CRC) is among the top three most common malignant tumors. According to statistical data for 2024, rectal cancer ranks 8th in terms of incidence among all malignant neoplasms in the world (1). In 20-25% of cases, after surgical treatment of CRC, patients experience disease progression in the form of metastases to the liver and lungs. These usually arise as a result of hematogenous spread. However, colorectal cancer often affects regional lymph nodes, which impacts 5-year survival rates and is one of the unfavorable prognostic factors (2).

According to medical literature data, isolated ureteral metastases are most commonly treated surgically. The available treatment options include nephroureterectomy (removal of the kidney and ureter), ureteral stenting, or ureteral resection (3, 4). Intestinal ureteroplasty in the context of colorectal cancer progression is a relatively rare surgical intervention. Only publications addressing the use of intestinal segments for partial or complete replacement of one or both ureters in other diseases of the urinary system have been presented (5, 6).

## Case presentation

Patient N., 48 years old, presented to Surgical Department No. 2 of University Clinical Hospital No. 4 at the I.M. Sechenov First Moscow State Medical University with clinical signs of rectal cancer progression, specifically compression of the middle third of the left ureter by the tumor. The patient had previously 4 years prior, the patient underwent radical surgery for malignant neoplasm of the rectum, pT2N0M0 Stage I, in the form of laparoscopic low anterior resection of the rectum with D2 lymph node dissection and the formation of a preventive transverse colostomy. Based on the results of the pathomorphological examination, adjuvant chemotherapy was not administered. Two months after the surgical treatment, the patient underwent restoration of intestinal continuity with closure of the transverse colostomy.

38 months later, upon re-evaluation, contrast-enhanced computed tomography (CT) of the abdominal cavity revealed a mass along the middle third of the left ureter with signs of invasion into the ureteral wall and the left psoas muscle. Additionally, heterogeneity in the structure of the left ovary was noted (**Figure 1**). These findings indicated progression of the primary disease in the form of isolated distant metastases.

**Figure 1:**
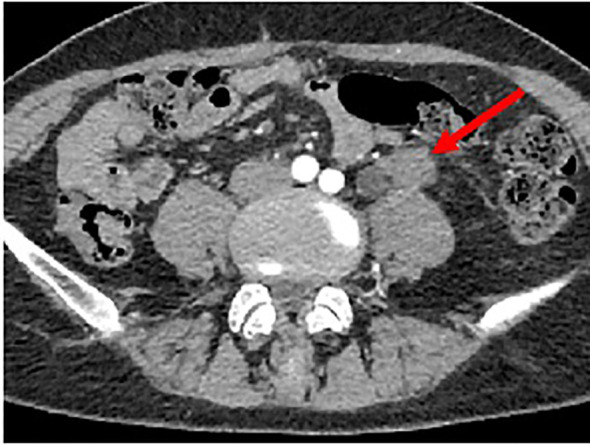
Preoperative CT image: axial; a mass along the middle third of the left ureter with the signs of invasion into the wall of the ureter itself and into the left lumbar muscle.

As part of the preoperative diagnostic workup, the patient underwent retrograde and antegrade pyeloureterography. The procedure revealed a 2 cm segment of non−opacification (lack of contrast filling) in the left ureter, localized at the level of vertebrae L4–L5. Given the extensive compression of the middle third of the left ureter at the tumor level, posing a threat to renal function, a percutaneous nephrostomy was performed. The renal filtration function was determined to be preserved.

The decision to perform surgical intervention was made at a multidisciplinary tumor board, in accordance with regional clinical guidelines, based on the stage of the oncological process, the patient’s satisfactory condition, and the absence of absolute contraindications.

Subsequently, surgical intervention was carried out, which included combined removal of the rectal cancer metastasis with resection of 10 cm of the left ureter, left-sided adnexectomy, and resection of the left colon that had been pulled down to the rectal stump. Intraoperatively, an extensive tumor conglomerate was identified, consisting of fatty tissue around the inferior mesenteric artery (IMA) and the mesentery of the descending colon. The lower edge of the conglomerate was closely adherent to the left psoas muscle. The left ovary appeared suspicious for secondary involvement: it was enlarged to 6 cm, firm, with a nodular surface, and a brownish color.

At the level of the tumor infiltration, lateral to the left common iliac artery, the left ureter was identified. It was involved in an extensive tumor conglomerate extending from the base of the IMA to the bifurcation of the common iliac artery. Intraoperatively, involvement of the entire middle third of the left ureter was confirmed, leading to its resection. Given the extent of the surgical intervention, the uncertainty of the tumor process, and the high risk of postoperative complications, it was decided to postpone the replacement of the ureter with a segment of the small intestine to a second stage, provided there was no further disease progression.

The morphological examination of the removed specimen revealed metastatic involvement of the ovary and the mesentery of the descending colon. Importantly, no signs of tumor invasion into the left ureter were found, but tumor deposits were located at a minimal distance (0.3-0.4 cm) from the ureteral wall.

Subsequently, the patient underwent 4 courses of adjuvant polychemotherapy according to the XELOX regimen. Follow-up examinations after the completion of chemotherapy showed no signs of disease progression.

Six months after the resection of the recurrent tumor, the patient had a repair of the left ureter with the small intestine segment, lowering of the transverse colon with the formation of a mechanical circular transversorectal end-to-end anastomosis, entero-enteroanastomosis, and preventive ileostomy.

## Surgical techniques

After adhesiolysis and incision of Gerota’s fascia, the following structures were sequentially isolated and mobilized from the pararenal fat: the lower and middle segments of the left kidney, the upper third of the ureter, and the renal pelvis. Subsequently, the apex and anterolateral left wall of the urinary bladder were exposed. Among the technical challenges encountered, it is important to note significant adhesions in the area of previous surgical interventions, which complicated the mobilization of the renal hilum structures and the urinary bladder.

To determine a suitable intestinal graft, a 30 cm segment of the small intestine was isolated 30 cm proximal to the ileocecal valve and transected. The vascular pedicle was preserved to ensure the viability of the graft (**Figure 2**).

**Figure 2:**
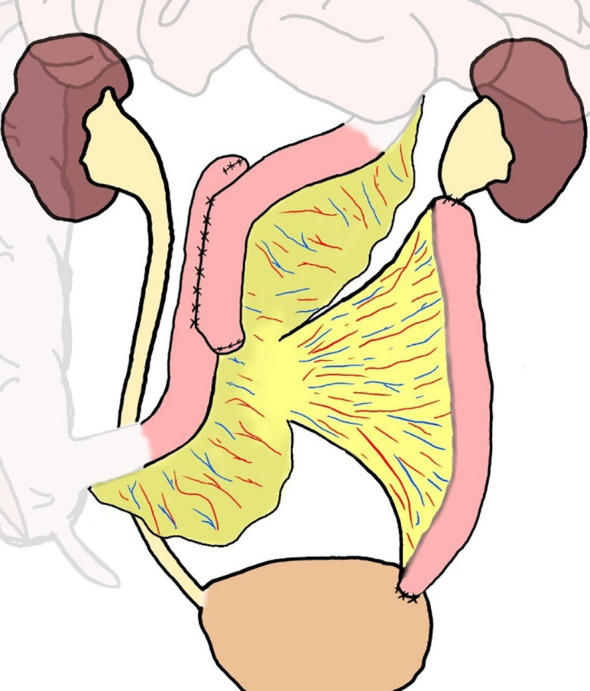
Scheme of left ureteral reconstruction with the small intestinal graft.

In the classical approach, the intestinal graft would have been passed through an opening in the colon mesentery retroperitoneally to the left renal pelvis. However, given the absence of the left colonic segments in the patient and the descent of the transverse colon, this step was not required. The segment of small intestine used for the graft was positioned in an isoperistaltic manner. Subsequently, an ileopyelostomy was created using a single-layer continuous Vicryl 3-0 suture, starting from the mesenteric edge.

The terminal transverse colostomy was closed, and a circular stapled transverse colorectal anastomosis was created.

The next step involved creating an anastomosis without an antireflux mechanism between the caudal end of the ileal graft and the urinary bladder. This was accomplished using two continuous opposing sutures with V-loc 3-0 suture material. The integrity of the urinary anastomoses was tested and confirmed to be competent (**Figure 3**).

**Figure 3:**
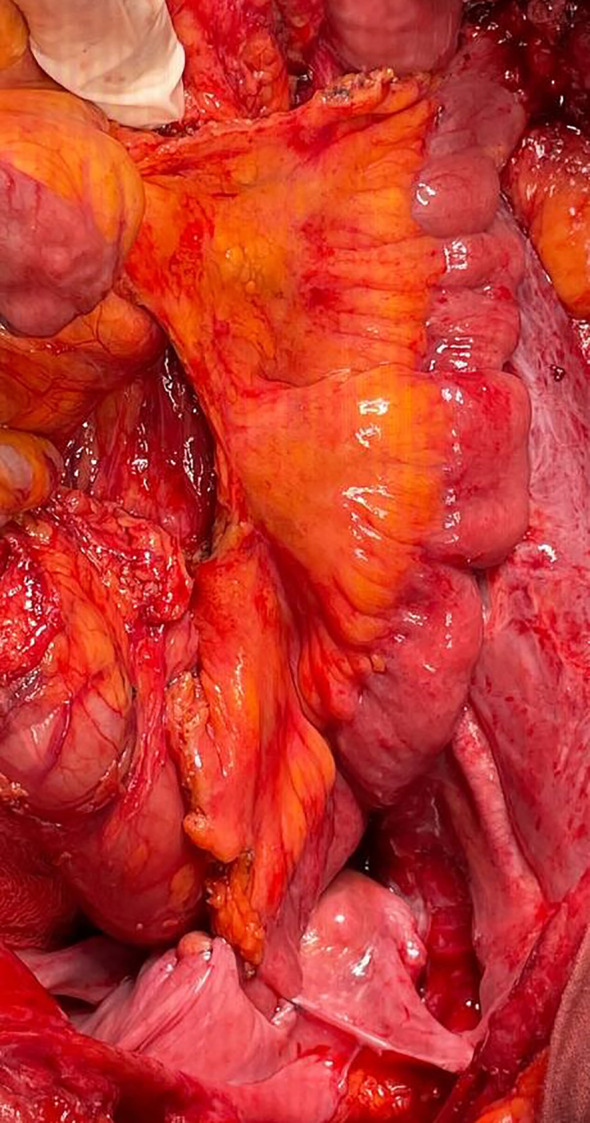
Final presentation of complete ureteral substitution using an intestinal segment (ileal ureter reconstruction).

The duration of the surgical intervention was 360 minutes; blood loss amounted to 200 ml.

Throughout the postoperative follow−up period, the patient’s condition remained stable and satisfactory, and she reported being satisfied with the treatment outcome.

Subsequently, the patient was discussed at a multidisciplinary oncology consultation, where it was decided not to proceed with adjuvant chemotherapy.

The total follow-up period since the last surgery is 32 months, with no evidence of disease progression. The patient is under regular dynamic observation. The depicted 3D-reconstruction of the left ureter, performed 32 months post-surgery, demonstrates complete opacification, confirming its patency and functional integrity (**Figure 4A**).

**Figure 4:**
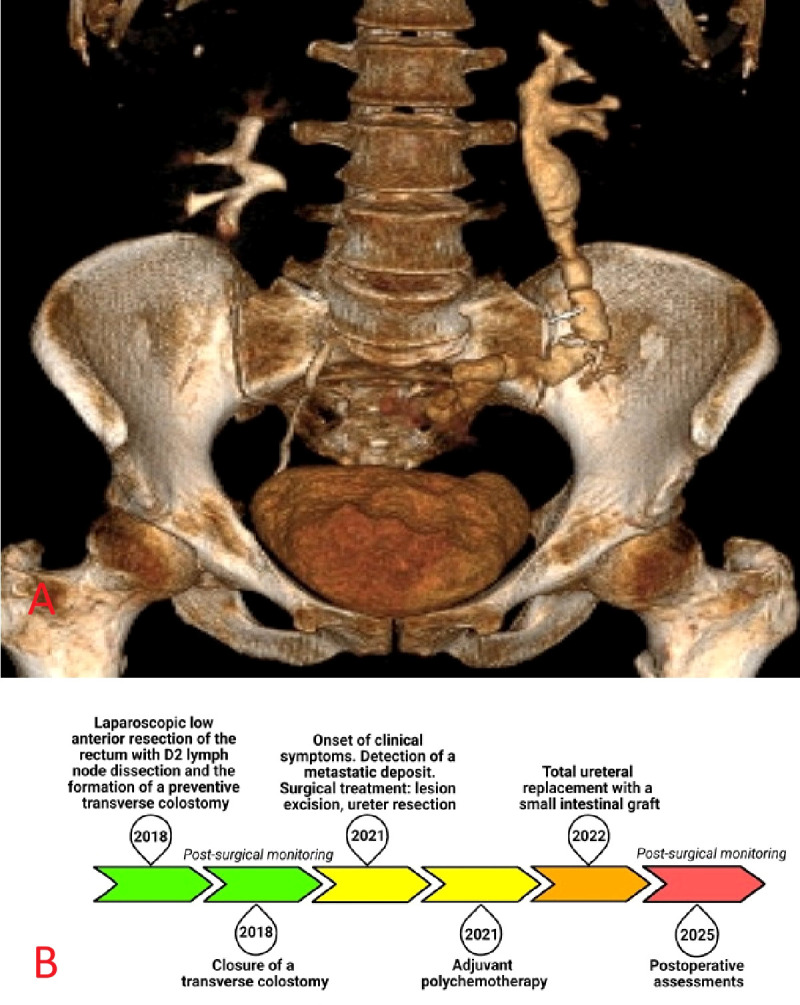
**(A)** 3D-reconstruction of the left ureter 32 months after the surgery. **(B)** Graphical representation of the patient’s clinical timeline.

This case demonstrates the possibility of cancer recurrence area of the apical lymph nodes following a previously performed D2 lymph node dissection. Ureteral reconstruction using intestinal segments in the context of colorectal cancer progression is a rarely performed surgical intervention. In this case, the total replacement of the left ureter with a segment of small intestine enabled the patient to maintain a decent quality of life. The procedure also eliminated the need for nephrostomy drainage, thereby preventing the deterioration of left kidney function that would inevitably occur with prolonged direct external drainage of the organ.

A graphical representation of the patient’s clinical timeline is illustrated in **Figure 4B**.

## Discussion

Currently, rectal cancer ranks 8th in the structure of malignant neoplasms in the world (1). A revolutionary breakthrough in the treatment of colorectal cancer, which significantly improved oncological outcomes, was the introduction of the principles of surgical dissection along embryonic layers. This innovative approach, first scientifically substantiated by Professor R.J. Heald in the development of total mesorectal excision (TME), involves the precise removal of the rectum within the mesorectal fascia. The introduction of TME marked a new era in the treatment of rectal cancer, delivering impressive results that surpass the survival rates for colon cancer (7). Subsequently, with the development of radiation therapy, chemotherapy, and the standardized implementation of total mesorectal excision, the 5-year survival rate after comprehensive treatment has reached 65.5-80.2% (8, 9). Despite the positive outcomes of multimodal therapy, tumor recurrence remains a significant challenge. With a median follow-up of up to 5 years, the recurrence rate is 4.7-8% (9). A crucial role is played by extended D3 lymph node dissection with the removal of the apical group of lymph nodes at the base of the inferior mesenteric artery. According to authors Hideki Ueno and Kenichi Sugihara, metastases to the apical lymph nodes occur in 2.8% of cases (10). In Japan, considering the low frequency of metastases to the apical lymph nodes and the sometimes-insufficient accuracy of preoperative diagnostics, D3 lymph node dissection has become a standard procedure for colorectal cancer with clinical stages cT3 and cT4, as well as in the presence of lymphadenopathy. Moreover, the guidelines of the Japanese Society for Cancer of the Colon and Rectum allow for D2 or D3 lymph node dissection in patients with a clinical picture of cT2 (7).

According to the results of a retrospective cohort study conducted by Wang L. et al., D3 lymph node dissection significantly improved survival in the surgical treatment of colorectal cancer (CRC) at stage pT2. The effectiveness of D3 lymph node dissection is most clearly demonstrated when comparing the outcomes of this technique with standard surgical intervention, where the extent of lymph node dissection is assessed as D2. In a comparison of two groups of patients treated at a major cancer center in Japan from April 2007 to December 2020, it was shown that the overall 5-year survival rate was significantly higher with D3 lymph node dissection compared to D2 lymph node dissection (p=0.036). Additionally, the recurrence-free survival rates after D3 lymph node dissection were significantly higher (p=0.013) (11).

Given the clinical picture of disease progression in the form of an isolated tumor process in the fatty tissue around the IMA, compression with tumor tissue invasion into the middle third of the left ureter, obstruction of the left upper urinary tract, and the development of ureterohydronephrosis, the indications for surgical treatment are absolute.

The selection of a reconstructive procedure for the upper urinary tract depends on multiple factors, including the specific location of the compromised ureteral segment (upper, middle, or lower third), the extent of stricture, obliteration, or external compression, as well as the anatomical characteristics of the renal pelvis system, such as its intra- or extrarenal location and accessibility for anastomotic creation.).

In reconstructive surgery of the upper urinary tract, native tissues are always preferred when technically feasible. In cases of deficiency in the lower third of the ureter, a direct ureteroneocystostomy with the Psoas hitch technique is possible (fixation of the bladder to the tendon of the psoas muscle just above the genitofemoral nerve using remnants of the peritoneum, or without fixation if a tension-free anastomosis can be achieved) (12). In cases of complete absence of the lower third, with or without partial deficiency of the middle third of the ureter, a bladder flap can be created and transformed into a neoureter (Boari flap or its modification). Typically, a Boari flap can compensate for a ureteral defect up to the level of the common iliac artery (13).

Deficiency of the upper third of the ureter can be compensated either by a direct end-to-end anastomosis or by creating a flap from a large extrarenal pelvis. The latter is usually possible in cases of classic hydronephrosis. However, in urolithiasis, the pelvis is typically small, intrarenal, or mixed, making it impossible to use a pelvic flap to compensate for the deficiency of the upper third of the ureter resulting from complications of surgical treatment for nephrolithiasis. In such cases, a large expanded lower calyx is sometimes utilized, where after removing the renal parenchyma of the lower segment of the kidney, it becomes possible to form an anastomosis between the ureter and the lower calyx (the Neivert procedure) (14).

Short strictures of the middle third of the ureter (up to 2-3 cm) can sometimes be compensated by end-to-end anastomotic plasty. For strictures of the middle third not exceeding 4-5 cm in length, a tubularized intestinal graft (the Monti procedure), appendix, or buccal graft may be used (15–17). The latter is quite promising, but for the viability of the graft, the initial preservation of the ureteral lumen is necessary. Tubularization of the buccal graft is not recommended due to the high risk of compromised blood supply.

For extensive ureteral strictures, obliterations, or external compressions, the procedure of choice is either total (complete organ replacement) or subtotal ileal ureteral reconstruction. The formation of anastomoses with wide hollow organs, such as renal pelvises and the urinary bladder, makes total ureteroileoplasty more preferable than subtotal ureteroileoplasty (where one end of the intestine is anastomosed with the middle or upper third of the ureter). It is important to note that with the subtotal modification, there is a higher likelihood of anastomotic narrowing at the ureteral junction.

It has been experimentally and clinically proven that harvesting 30-40 cm of the small intestine for substitutive ureteral plasty does not result in clinically significant water-electrolyte imbalances (18).

Despite the fact that ureteroileoplasty has been increasingly used in recent years in reconstructive surgery of the upper urinary tract, the procedure is considered technically complex. The main disadvantages of open substitutive small intestinal ureteroplasty via laparotomy access are the traumatic nature of the procedure and the difficulty in accessing the pelvis due to the deep location of the kidney. The situation is exacerbated during surgery on the left side and in cases of a small intrarenal pelvis. Therefore, laparoscopic ureteroileoplasty, despite the multiquadrant access and complex technical equipment, is sometimes preferable for both the patient and the surgeon. The main contraindication for laparoscopic access is the presence of multiple abdominal adhesions, which significantly prolong and increase the risks of the surgical intervention (19, 20).

The most physiological position for the ureteroileal transplant is considered to be as close as possible to the native ureter. On the left side, the ileoureter is usually conducted through the mesentery of the large intestine. On the right side, with a high hepatic flexure of the ascending colon, a similar technique is possible, when the graft passes behind the cecum. It is necessary to maintain isoperistaltic positioning of the ureteroileal graft. For normal functioning of the intestinal graft and restoration of urodynamics, the latter must have adequate blood supply and be maximally “verticalized” without significant bends.

Substitutive small intestinal ureteroplasty becomes an indispensable option for extensive ureteral strictures in cases of clinically significant chronic kidney disease, where kidney removal is associated with the risk of transitioning the patient to an anephric state. Prolonged use of nephrostomy drains, as well as internal drainage, is associated with a gradual irreversible deterioration of kidney function (21, 22).

According to WHO statistics, the need for simultaneous surgeries arises in approximately 30% of all surgical patients. Thus, substitutive ureteroileoplasty can be highly appropriate and effective in the interdisciplinary field under certain suitable circumstances.

The question of choosing between D2 and D3 lymph node dissection for rectal cancer at stage pT2N0M0 remains open. The guidelines of the Japanese Society for Cancer of the Colon and Rectum (JSCCR) consider both options of lymph node dissection – D2 or D3 – for patients with clinical stage cT2. However, in this patient, after D2 lymph node dissection, disease progression was observed in the fatty tissue around the IMA, which is only removed during D3 lymph node dissection. This clinical case highlights the appropriateness of performing D3 lymph node dissection for rectal cancer even with a small tumor size and preoperative staging not exceeding T2N0M0.

In conclusion, total replacement of the ureter using an intestinal graft is regarded as the optimal reconstruction method for the upper urinary tract in patients with progressive colorectal cancer. In the presented clinical case, this surgical intervention ensured proper restoration of urinary drainage from the kidney, maintained renal function, and contributed to both improved quality and prolonged duration of the patient’s life. Additionally, the possibility of recurrence in the fatty tissue around the IMA justifies the advisability of D3 lymph node dissection, even in cases of small tumors.

## Data Availability

The original contributions presented in the study are included in the article/supplementary material. Further inquiries can be directed to the corresponding author.
